# Targeting Bacterial Dsb Proteins for the Development of Anti-Virulence Agents

**DOI:** 10.3390/molecules21070811

**Published:** 2016-07-16

**Authors:** Roxanne P. Smith, Jason J. Paxman, Martin J. Scanlon, Begoña Heras

**Affiliations:** 1 Department of Biochemistry and Genetics, La Trobe Institute for Molecular Science, La Trobe University, Kingsbury Drive, Bundoora, Vic 3083, Australia; rp2smith@students.latrobe.edu.au (R.P.S.); J.Paxman@latrobe.edu.au (J.J.P.); 2 Medicinal Chemistry, Monash Institute of Pharmaceutical Sciences, Monash University, Royal Parade, Parkville, Vic 3052, Australia; martin.scanlon@monash.edu

**Keywords:** *disulfide catalysis*, DsbA inhibitors, DsbB inhibitors, anti-virulence, fragment-based drug design, antimicrobial resistance

## Abstract

Recent years have witnessed a dramatic increase in bacterial antimicrobial resistance and a decline in the development of novel antibiotics. New therapeutic strategies are urgently needed to combat the growing threat posed by multidrug resistant bacterial infections. The Dsb disulfide bond forming pathways are potential targets for the development of antimicrobial agents because they play a central role in bacterial pathogenesis. In particular, the DsbA/DsbB system catalyses disulfide bond formation in a wide array of virulence factors, which are essential for many pathogens to establish infections and cause disease. These redox enzymes are well placed as antimicrobial targets because they are taxonomically widespread, share low sequence identity with human proteins, and many years of basic research have provided a deep molecular understanding of these systems in bacteria. In this review, we discuss disulfide bond catalytic pathways in bacteria and their significance in pathogenesis. We also review the use of different approaches to develop inhibitors against Dsb proteins as potential anti-virulence agents, including fragment-based drug discovery, high-throughput screening and other structure-based drug discovery methods.

## 1. Introduction

The dramatic increase in the rate of antimicrobial resistance, especially in the last two decades, is a worldwide health concern [1] and has been made an international health priority by the World Health Organization (WHO) [2]. Since the serendipitous discovery of penicillin in the 1940s, bacteria have developed resistance to all antibiotics introduced into clinical practice [3]. This rise in antibiotic resistance is exacerbated by a decline in the development of novel antibiotics [4,5]. We are on the verge of entering a “post-antibiotic era” where previously treatable common infections may no longer be effectively treated with antibiotics. If no immediate action is taken, by 2050 the annual mortality rate associated with multi drug resistant (MDR) pathogens is predicted to surpass that of cancer, with 10 million deaths annually [6]. Therefore, new approaches are urgently needed to tackle the dramatic escalation of antibiotic resistant infections [7,8].

A possible antibacterial strategy is to develop compounds that inhibit bacterial virulence rather than bacterial growth [9,10,11]. Pathogens rely on an arsenal of virulence factors, such as fimbrial and non-fimbrial adhesins, toxins, Type 3 secretion systems (T3SS) and motility organelles, to attach and infect their host. Anti-virulence strategies focus on disarming bacteria of these virulence systems thereby reducing or completely abolishing their capacity to cause disease (reviewed in [12]). This strategy offers several advantages including broadening the repertoire of antimicrobials and also potentially reducing the selection pressure for the development of bacterial resistance [13]. 

In this review we focus on a central system that regulates the deployment of multiple virulence factors in bacteria, the disulfide bond (Dsb) oxidative folding machinery. We describe disulfide bond catalysis pathways in bacteria and how these pathways affect bacterial pathogenesis. We also review the advances made in targeting these redox systems for the development of anti-virulence agents.

## 2. Dsb Disulfide Forming Pathways in the Model Organism *E. coli* K-12

Disulfide bonds between pairs of cysteine residues confer stability to secreted and surface exposed proteins, which include numerous bacterial virulence factors [14]. In bacteria, this process is mediated by the Dsb family of proteins [15]. Dsb enzymes have been best characterized in *Escherichia coli* K-12 [16,17] where they form two separate pathways; an oxidative pathway which introduces disulfide bonds into folding proteins, and an isomerase pathway which corrects non-native disulfide bonds [18].

### 2.1. Dsb Oxidative Pathway

**Figure 1:**
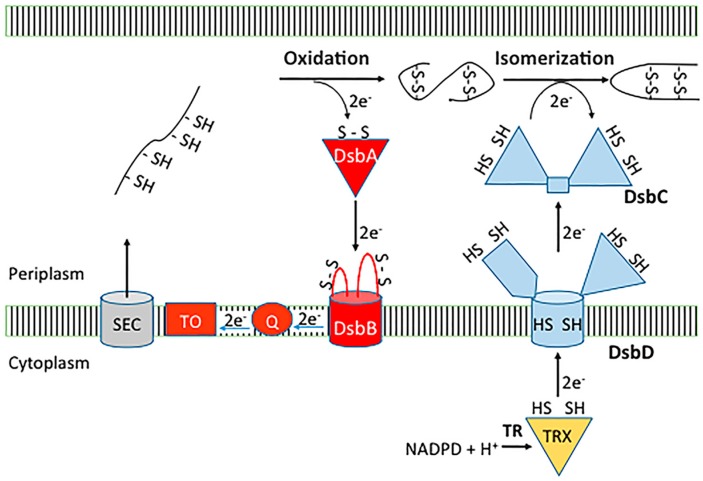
*Escherichia coli* K-12 disulfide catalytic pathways. In the oxidase pathway the thioredoxin-like oxidase DsbA introduces disulfide bonds into proteins that are translocated to the periplasm via the SEC machinery (the plotted line with the -SH and S-S symbols represents the amino acid chain of the DsbA substrate protein). Upon oxidising a substrate, DsbA becomes reduced and is re-oxidized by the partner membrane protein DsbB, which transfers electrons to quinones (Q) and terminal oxidases (TO). In the isomerase pathway, incorrectly formed disulfide bonds are corrected by the isomerases DsbC and DsbG, which are maintained in a reduced form by the inner membrane reductase DsbD. This multidomain protein is reduced by cytoplasmic thioredoxin, which in turn is reduced by thioredoxin reductase (TR) in a NADPH-dependent manner.

**Figure 2:**
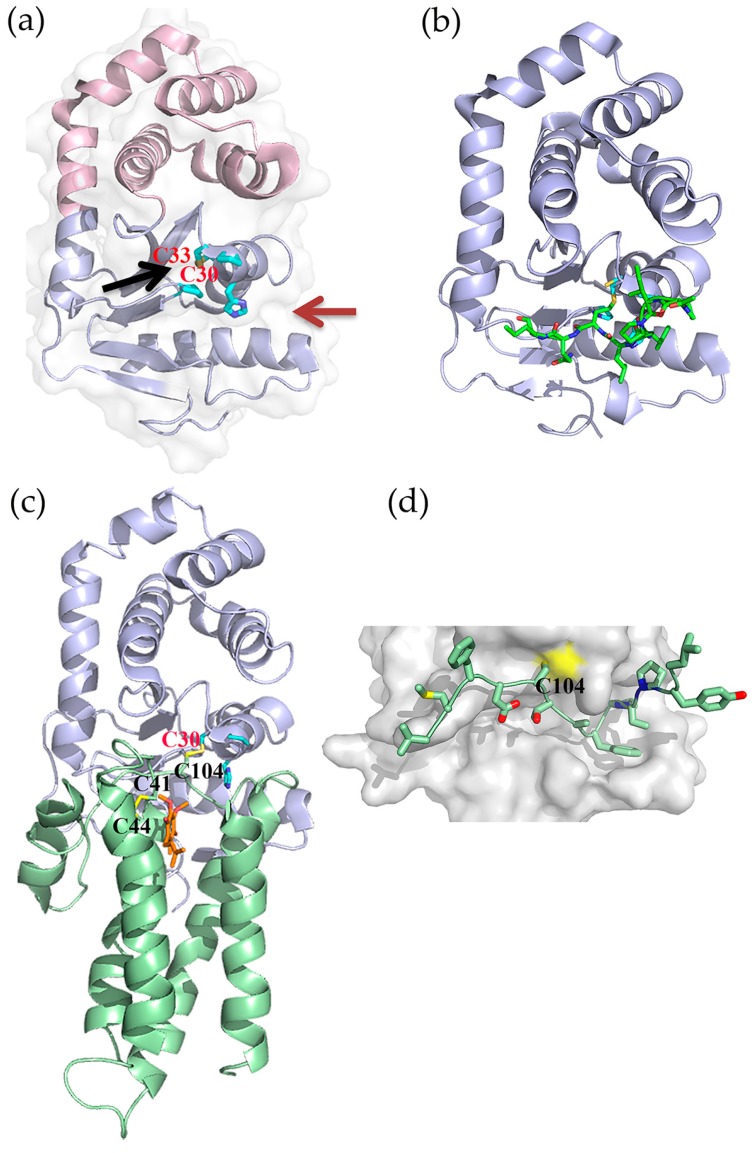
(**a**) Cartoon representation of EcDsbA (PDB 1FVK); thioredoxin fold shown in light blue and helical insert in light pink. Red and black arrows indicate the hydrophobic groove and hydrophobic patch, respectively; (**b**) Substrate peptide binding surface of EcDsbA (PDB 3DKS). Peptide and enzyme are shown in green and light blue respectively; (**c**) Crystal Structure of the EcDsbA–EcDsbB–UQ complex (PDB 2HI7). EcDsbA and EcDsbB are shown in cartoon representation (light blue and green respectively). DsbA Cys30 and DsbB Cys41,44, and 104 are displayed in stick representation. UQ molecule bound to DsbB is displayed in stick representation (orange); (**d**) Close-up view of the DsbB loop interaction site with the hydrophobic groove of EcDsbA. The DsbA the active site residues (Cys30-Pro-His-Cys33) and *cis*-proline residue are displayed in stick representation (cyan) in panels (**a**) to (**c**).

In *E. coli* K-12 the oxidative pathway comprises two Dsb catalysts, DsbA (EcDsbA) and DsbB (EcDsbB) (Figure 1). When proteins enter the periplasm DsbA introduces disulfide bonds between pairs of cysteine residues [19,20]. The structure of EcDsbA comprises a thioredoxin-like domain with an inserted helical domain containing a three helical bundle and two additional α-helices [21] (Figure 2a). Like other thiol oxidase enzymes, DsbA has the characteristic CXXC (Cys30-Pro31-His32-Cys33 in EcDsbA) redox active site flanked by a hydrophobic groove and a large hydrophobic patch [21,22]. The CXXC active site, hydrophobic patch and a highly conserved *cis*-proline (Pro151 in EcDsbA) loop, are important for substrate binding [22,23] (Figure 2b). Substrate binding is mediated by backbone-to-backbone hydrogen bonds with residues of the *cis*-proline loop, which allows many DsbA enzymes to exhibit broad substrate specificity.

Oxidation of substrate proteins leaves the catalytic site of DsbA reduced. Subsequently, the cytoplasmic membrane protein DsbB re-oxidizes DsbA [20,24]. DsbB binds to DsbA in a similar mode to its substrate proteins, but additional interactions occur within the hydrophobic groove [25]. EcDsbB consists of four transmembrane α-helices arranged into a bundle configuration and linked by one cytoplasmic and two periplasmic loops [25] (Figure 2c). Each periplasmic loop contains a pair of redox active cysteine residues (Cys41/44 and Cys104/130) and in its oxidized state both pairs exist in their disulfide bonded form [26]. Reoxidation of EcDsbA by EcDsbB proceeds via a mixed disulfide complex with a disulfide formed between the Cys104 of DsbB and the Cys30 of DsbA [11] (Figure 2d). Subsequently, a disulfide-dithiol exchange cascade proceeds in which DsbB Cys41/44 reoxidises Cys104/130 [27,28] and Cys41/44 is re-oxidised by ubiquinone/menaquinone that is bound to DsbB [29,30].

### 2.2. Dsb Isomerase Pathway

DsbA can introduce non-native disulfide bonds into proteins that contain more than two cysteines [31,32]. Disulfide bonds are corrected by DsbC and to a lesser extent DsbG disulfide bond isomerases within the periplasm [18,33,34] (Figure 1). Both DsbC and DsbG are V-shaped homo-dimers, with each monomer containing a C-terminal thioredoxin domain with the characteristic CXXC redox motif [35,36]. The isomerase activity depends upon the maintenance of DsbC/DsbG in a reduced state [37], which is mediated by the cytoplasmic membrane protein DsbD [34,37]. DsbD is a multidomain protein consisting of two periplasmic domains (n-DsbD and c-DsbD) and a central transmembrane domain (t-DsbD) [38]. Structurally, n-DsbD has an immunoglobulin-like fold [39], c-DsbD has a thioredoxin-like fold [40] and the central t-DsbD is an integral membrane domain consisting of 8-transmembrane helices [41]. A succession of disulfide bond exchange reactions transfers electrons from cytoplasmic thioredoxin [37,41], to each pair of catalytic cysteines within the t-DsbD, c-DsbD and n-DsbD, which allows DsbD to reduce its periplasmic substrates DsbC and DsbG [39].

## 3. Distribution of Dsb Systems across Bacteria

Much of our current knowledge of bacterial disulfide catalysis has derived from studies carried on *E. coli* K-12. A clearer understanding of the diversity of disulfide catalysis throughout bacteria has emerged from the ever-increasing number of whole prokaryotic genome sequences, which show that Dsb enzymes, particularly DsbA homologues, are present in most bacteria [14,17,42]. However, the K-12 paradigm of Dsb folding enzymes that form two separate pathways is only conserved in Gamma- and Beta-Proteobacteria. Despite the Dsb pathway conservation in these bacterial classes, some variation is observed in the number and type of Dsb proteins. For example, the uropathogenic *E. coli* (UPEC) strain CFT073, which is closely related to *E. coli* K12, contains both the DsbA/DsbB oxidase as well as an additional DsbL/DsbI redox pair, which may be dedicated to a select group of substrates [43]. Other organisms have also been reported which contain an extended number of Dsb proteins. For example, some *Salmonella enterica* serovars contain the prototypic *E. coli* K-12 oxidase and isomerase systems as well as the DsbL/DsbI pair and a virulence plasmid-encoded DsbA-like protein, called SrgA [44,45]. *Neisseria meningitidis* also has both the oxidase and isomerase systems but without DsbG, as well as two additional DsbA-like lipoproteins anchored to the inner membrane [46,47]. In contrast, bacteria from other groupings typically have a reduced number of Dsb catalysts [14]. For example, Alpha-, Delta- and Epsilon-Proteobacteria usually lack all enzymes in the isomerase pathway [14]. Similarly, Gram-positive bacteria such as *Staphylococcus aureus* and *Listeria monocytogenes* only encode a DsbA but they do not encode any other Dsb protein [48].

The most taxonomically widespread Dsb protein is DsbA, which is found in all classes of Proteobacteria and Chlamydiales along with numerous species of Fusobacteria and Actinobacteria [14]. Despite low sequence homology, DsbAs share a conserved three-dimensional architecture (reviewed in [17]). A recent comparative analysis of well characterised DsbAs from different bacteria showed that these proteins can be assigned into two groups, referred to as DsbA-I and DsbA-II, which differ primarily on the central β-sheet topology in the TRX-fold [49]. These groups can be further subdivided into the subgroups Ia, Ib, IIa and IIb, on the basis of structural and redox features [49]. Type Ia DsbAs contain the largest DsbB binding grooves and are mostly from Enterobacteriaceae, which includes EcDsbA. The Type Ib DsbAs from Beta- and Gamma-Proteobacteria contain smaller binding pockets and are more oxidising than the Type Ia DsbAs. The Type II DsbAs largely from Gram-positive bacteria exhibit highly charged electrostatic surfaces around the active site and less defined binding pockets [49]. The division of DsbA homologues into structural subclasses that are broadly associated to different classes of bacteria may provide a basis for developing inhibitors with a DsbA-subclass spectrum of activity [49]. This is highly relevant given the increasing body of work linking disulfide catalysts to bacterial pathogenesis [14,50,51].

## 4. Dsb Systems and Bacterial Virulence

**Figure 3:**
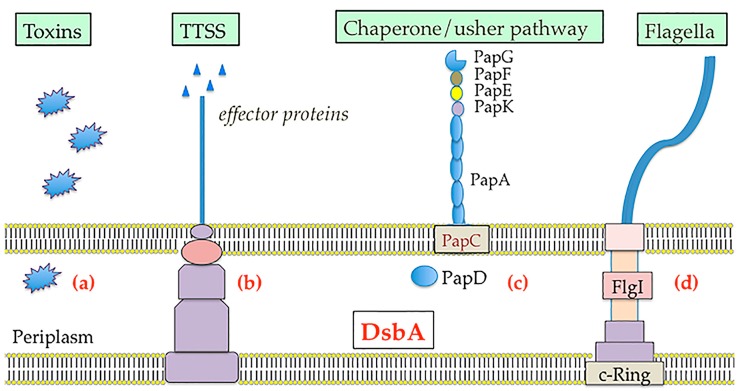
Overview of selected DsbA dependent virulence factors: (**a**) secretion of toxins; (**b**) assembly of the type III secretion system (TTSS); (**c**) folding of different components of the usher chaperon system for fimbriae biosynthesis (e.g., P fimbriae); (**d**) assembly of the flagellar motor (FlgI). Detailed description of the virulence mechanisms dependent on the DsbA/DsbB system can be found in other reviews [14,17].

Bacterial cells containing *dsbA* null mutations show a pleiotropic phenotype due to the incorrect folding of many periplasmic proteins (alkaline phosphatase, β-lactamase and OmpA among others) and reduced fitness in animal models [52,53,54,55]. Furthermore, they display attenuated virulence since the folding, stability and function of many bacterial virulence factors including toxins, secretion systems, adhesins and motility machines are dependent upon DsbA/DsbB mediated disulfide bond formation (Figure 3) (reviewed in [14], [17]). For example, *V. cholerae*, enteropathogenic *E. coli* and *Bordetella pertussis dsbA* mutants secrete reduced levels of cholera, heat-labile and pertussis toxins respectively [56,57,58]. In most cases the reduced levels of toxin subunits within cellular extracts, suggests that in the absence of DsbA catalysed disulfide bond formation the toxin subunits cannot fold into their native structures.

Similarly, the delivery of virulence factors can also be dependent upon DsbA-mediated disulfide bond formation. Many bacterial pathogens use T3SS to inject effector proteins into the cytosol of host cells to modulate eukaryotic cell pathways [59,60]. The outer membrane secretins within the T3SS of *Shigella flexneri* [61], *Yersinia pestis* [62] and *Salmonella* Typhimurium [63] among others require an intramolecular disulfide bond for correct folding. As a result, in the absence of DsbA the T3SS of these pathogens is defective for transporting effector proteins.

DsbA is also required for the production or stability of adhesins such as Type 4 fimbriae and pili, which are important for bacterial colonisation. For example, the major structural subunit BfpA of the bundle-forming pilus of enteropathogenic *E. coli* (EPEC) requires a DsbA mediated intramolecular disulfide bond [64]. Similarly, the P pili chaperone PapD cannot adopt a stable structure without its disulfide bond added by DsbA, which prevents pili subunit assembly on uropathogenic *E. coli* [65]. Flagella mediate motility and the spread of pathogenic bacteria. DsbA catalyses disulfide bond formation within the FlgI flagellar P-ring motor protein [66]. As such a mutation in *dsbA* or *dsbB* has been shown to render *E. coli* along with many other bacteria such as *Salmonella enterica* [67] and *Proteus mirabilis* [68] non-motile. 

Clearly, depletion of the DsbA/B thiol oxidation system has pleiotropic effects on multiple virulence-associated phenotypes and also diminishes the capacity of pathogens to establish infections in animal models. For example, mice infected with *Burkholderia pseudomallei* only survived when infected with a *dsbA* deletion strain [69]. Similarly, deletion of *dsbA/dsbB* in UPEC significantly reduced colonisation of the bladder in a mouse model of infection [55]. *Salmonella enterica* Typhimurium *dsbA* deletion mutants were also significantly attenuated in a murine infection model [63].

## 5. Targeting Dsb Proteins for the Development of Anti-virulence Agents

The widespread distribution of the DsbA/DsbB redox system across bacteria and the reliance of many virulence factors on DsbA mediated disulfide bond formation, makes this oxidative system an attractive candidate to target for the development of anti-virulence therapeutics. However, inhibiting these enzymes has a number of challenges. Finding suitable binding sites on DsbA–substrate protein binding interfaces presents the greatest challenge. The most stabilizing interaction DsbA forms with the majority of its substrates, is mediated via a disulfide bond formed by the nucleophilic cysteine (Cys30 in EcDsbA), but this bond is rapidly resolved as a result of the reactivity of this cysteine [70]. The integral membrane protein DsbB also presents a significant challenge due to the technical difficulties associated with working with a membrane protein outside of its native environment. However, in the last 5 years there have been several publications reporting different approaches to targeting the DsbA/B oxidative pathway. This review highlights the different approaches used, which can be loosely grouped into inhibition of DsbA/DsbB with chemical fragments; inhibition of DsbA with peptides and peptidomimetics; and high-throughput screening of chemical compounds against DsbB. 

### 5.1. Fragment-Based Drug Discovery Targeting E. coli DsbB 

Fragment-based drug discovery (FBDD) has emerged as a powerful strategy for developing leads against a wide variety of drug targets and has already yielded compounds that are approved for clinical use [71,72].

**Figure 4:**
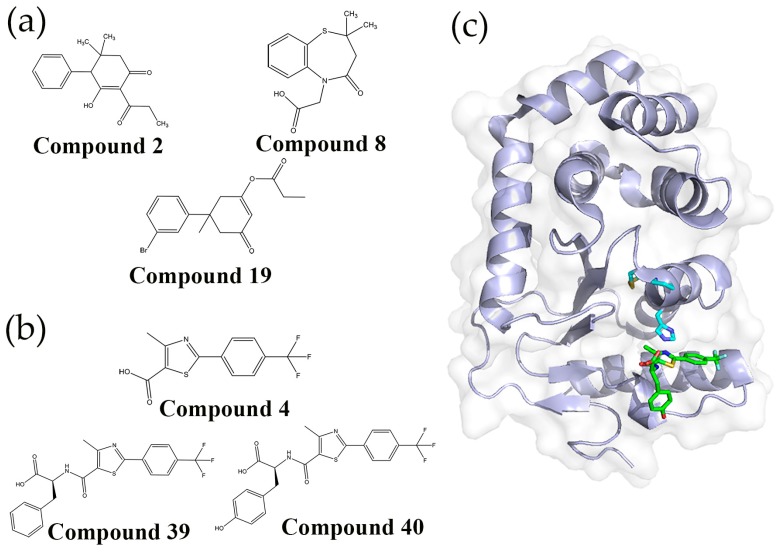
(**a**) Potent DsbB inhibitors identified by a fragment-based drug discovery (FBDD) method. Compound **2** (6-hydroxy-2,2-dimethyl-5-propionyl-2,3-dihydro-[1,1′-biphenyl]-4(1*H*)-one) has the same binding site as UQ1, compound **8** (2-(2,2-dimethyl-4-oxo-3,4-dihydrobenzo[*b*][1,4]thiazepin-5(2*H*)-yl)acetic acid) competes with both quinone and EcDsbA for binding EcDsbB. Compound **19** (3′-bromo-1-methyl-5-oxo-1,2,5,6-tetrahydro-[1,1′-biphenyl]-3-yl propionate) is the small molecule DsbB inhibitor developed from compound **2**; (**b**) Structures of compound **4** (4-Methyl-2-(4-(trifluoromethyl)phenyl)thiazole-5-carboxylic acid) the most potent DsbA inhibitor identified by a FBDD method, and a phenylalanine derivative (compound **39**, 4-Methyl-2-(4-(trifluoromethyl)phenyl)thiazole-5-carbonyl)-l-phenylalanine) and a tyrosine derivative (compound **40**, 4-Methyl-2-(4-(trifluoromethyl)phenyl)thiazole-5-carbonyl)-l-tyrosine); (**c**) Co-crystal structure of compound **40** bound to EcDsbA (PDB 4WET). The CPHC active site and *cis*-Pro residue are displayed in stick representation (cyan). Compound **40** is shown in stick representation with carbon atoms colored green.

One of the first attempts to identify anti-virulence agents that inhibit Dsb proteins involved using a FBDD approach to target the membrane disulfide oxidase EcDsbB [73]. It represented one of the first examples of FBDD against an integral membrane protein, which due to their physiochemical properties are less amenable to the biophysical screening techniques typically used for detecting fragment binding. The screening was undertaken using the “Target Immobilized NMR Screening” (TINS) technique. This technique involved solubilising EcDsbB into detergent micelles, immobilizing them onto sepharose resin, and then placing them into a flow-injection dual cell sample holder together with a reference for NMR screening. Fragment mixtures were simultaneously injected to both target and reference samples and hit fragments from a library of 1071 fragments were detected by comparing the 1D^1^H NMR spectra recorded for EcDsbB to the reference samples [73]. This approach yielded 93 hits, which were validated using an enzyme inhibition assay measuring the capacity of EcDsbB to reoxidize its substrate EcDsbA or reduce its cofactor Ubiquinone-5. A total of 8 fragments were found to significantly inhibit EcDsbB activity giving IC_50_ values from 7–170 μM*.* These fragments seemed to follow either of two modes of inhibition as shown by: (i) some fragments only competing with quinone for binding EcDsbB (compound **2**, (6-hydroxy-2,2-dimethyl-5-propionyl-2,3-dihydro-[1,1′-biphenyl]-4(1*H*)-one)) (Figure 4a); or (ii) other fragments competing with both quinone and EcDsbA for binding EcDsbB (compound **8**) (Figure 4a). These two different modes of interaction with EcDsbB were further confirmed using heteronuclear single quantum coherence (HSQC) spectroscopy using ^15^N-labelled protein.

From this study Früh *et al.* not only proved that NMR-based FBDD approaches are suitable to identify fragments targeting integral membrane proteins but also identified several chemical scaffolds for the development of specific DsbB inhibitors. In this context, a separate study by Halili and co-workers reported structure-activity relationship (SAR) studies performed on compound **2** (Figure 4a) for the development of more potent DsbB inhibitors [74]. The 2-propionoyl, 4-phenyl, and the two 5-methyl groups were stripped from the compound leaving the 1,3-cyclohexanedione. Substituents were progressively added back and tested for their effects on inhibiting Dsb mediated disulfide bond formation using a synthetic peptide substrate. Improvements in the IC_50_ values from 2000 μM to 1.1 μM were made with the additions of dimethyl, propionate and bromo-phenyl substituents to create compound **19** (3′-bromo-1-methyl-5-oxo-1,2,5,6-tetrahydro-[1,1′-biphenyl]-3-yl propionate) (Figure 4a). Addition of compound **19** to EcDsbB followed by denaturing liquid chromatography–mass spectrometry (LC-MS/MS) showed that it had left a stable 56 Da adduct on Cys130. Similarly, when compound **19** was incubated with reduced EcDsbA, analysis by electrospray ionization mass spectrometry (ESI-MS) showed that it added a 56 Da moiety onto Cys33. In contrast, analysis of ^1^H-^15^N HSQC data revealed that compound **19** was able to bind to oxidized EcDsbA, but there was no evidence of covalent modification of the oxidized enzyme. This data suggested that the developed compound inhibited both DsbA and DsbB enzymes through either a non-covalent interaction with the oxidized proteins or via the covalent addition of a propionyl group to one of the reduced active site cysteines. This work provides the first example of a small molecule with dual activity on DsbA and DsbB, The compound also showed a degree of selectivity as it was unable to inhibit human thioredoxin.

### 5.2. Fragment-Based Drug Discovery Targeting E. coli DsbA 

A FBDD approach has also been taken to identify fragments that bind non-covalently and inhibit EcDsbA [75]. Saturation transfer difference (STD) NMR was used in an initial screen to identify compounds from a library of 1132 fragments (Maybridge Ro3) that bound EcDsbA. The resulting 171 fragments were further validated by measuring ^15^N chemical shifts perturbations (CSP) of EcDsbA backbone amides in HSQC spectra upon addition of the fragments. This approach yielded 37 EcDsbA binders, the majority of which could be clustered into 5 different chemical classes. Analogues of the phenylthiazole class were further investigated as they showed interpretable SAR and were found by X-ray crystallography to bind the hydrophobic groove of EcDsbA, which is required for interacting with EcDsbB. Initially 22 commercial phenylthiazole analogs were assessed by the magnitude of chemical shift perturbations (CSP) produced in ^1^H-^15^N HSQC spectra of EcDsbA, which showed that 2-phenylthiazoles with halogen substituents on the aryl ring gave the greatest CSP. The co-crystal structure of one of these compounds 4-methyl-2-(4-(trifluoromethyl)phenyl)thiazole-5-carboxylic acid (compound **4**) (Figure 4b) with EcDsbA revealed notable interactions with residues in the EcDsbA hydrophobic groove and guided the development of additional synthetic analogues. Amino acid derivatives of compound **4** had the highest binding affinity for EcDsbA with *K*_D_ values in the 200–400 μM range, as determined by SPR. The phenylalanine (compound **39**) and tyrosine (compound **40**) derivatives (Figure 4b) were also found to inhibit EcDsbA activity *in vitro* with IC_50_ values of 185 ± 10 and 310 ± 16 μM, respectively (Figure 4b). Importantly, even at high-micromolar potency, compound **40** was able to inhibit motility in *E. coli* K-12 cells in a concentration-dependent manner, whilst having no effect on bacterial growth. This phenotype is consistent with inhibition of DsbA, which is required for the production of functional flagella [76] but is not essential for growth. The atomic resolution structure of EcDsbA in complex with compound **40** revealed that it occupies the hydrophobic grove of EcDsbA and displays an increased number of interactions with residues neighboring the active site compared to compound **4**, however the tyrosine group was unexpectedly found outside the EcDsbA hydrophobic groove (Figure 4c).

### 5.3. Peptides and Peptidomimetics Targeting DsbA 

**Figure 5:**
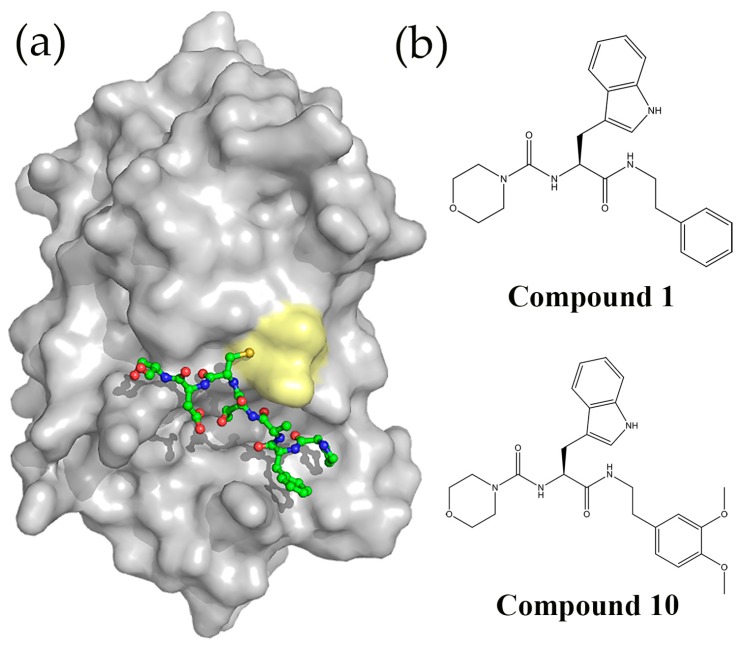
(**a**) Crystal structure of EcDsbA (depicted in surface representation) in complex with the EcDsbB derived peptide PFATCDS (shown in ball-and-stick representation) (PDB 4TKY). The EcDsbA active site is colored yellow; (**b**) Chemical structures of a two peptidomimetic compounds that inhibit DsbA. The left panel shows the top hit obtained from the virtual screening of library of peptidomitetics (compound **1**, (S)-*N-*(3-(1*H-*indol-3-yl)-1-oxo-1-(phenethylamino)propan-2-yl)morpholine-4-carboxamide). The right panel shows a synthesized peptidomimetic that inhibited EcDsbA activity at millimolar concentration (compound **10**, (*S*)-*N-*(1-((3,4-dimethoxyphenethyl)amino)-3-(1*H-*indol-3-yl)-1-oxopropan-2-yl)morpholine-4-carboxamide).

Additional efforts to discover DsbA inhibitors have involved developing peptides [77] and peptidomimetics [78] that prevent the formation of the DsbA-DsbB redox complex. Guided by the crystal structure of this complex [25] (Figure 2d), Duprez *et al.* designed a synthetic peptide (PFATCDS) that mimicked the DsbB periplasmic loop involved in DsbA docking (Figure 5a). This heptapeptide was found to bind EcDsbA with a *K*_D_ of 13.1 ± 0.4 µM. The EcDsbA/PFATCDS crystal structure revealed that the similarity to DsbB binding also included the peptide occupying the EcDsbA hydrophobic groove [77]. This structure was then used to design a series of peptides. Binding to DsbA was characterised using isothermal titration calorimetry (ITC) and inhibitory activity using a substrate oxidation assay, which led to identification of the peptide PWATCDS, which bound EcDsbA with a *K*_D_ of 5.7 ± 0.4 μM and inhibited its oxidase activity with an IC_50_ of 8.8 ± 1.1 μM. This peptide possessed some specificity towards DsbA as shown by its inability to bind or inhibit the structurally related human thioredoxin. Alanine scanning mutagenesis revealed that the cysteine residue was critical for binding to and inhibiting EcDsbA, which led the authors to propose that PWATCDS could be a more effective inhibitor if developed as an irreversible EcDsbA inhibitor. In a separate study, the same research group showed that the same peptide PWATCDS was also able to bind to the structurally similar *Proteus mirabilis* DsbA (PmDsbA) (another class Ia DsbA), with a *K*_D_ of 8.3 ± 0.4 μM [79]. They reported the crystal structure of a non-covalent complex between PWATCDS and the PmDsbAC30S mutant, which was stabilized by hydrogen bonding to the DsbA *cis*-proline loop and hydrophobic interactions with the DsbA hydrophobic groove. The possibility of exploiting these interactions for the development of non-covalent DsbA inhibitors, was further pursued using PWATCDS as a template for virtual screening of a peptidomimetic library [78]. The top hit along with nine derivatives were synthesized and found to inhibit EcDsbA at millimolar concentrations (Figure 5b). These peptidomimetics represented potential scaffolds for the elaboration of non-covalent DsbA inhibitors that will lack the limitations commonly associated with peptides such as lower stability, cell permeability and/or bioavailability. 

### 5.4. High-throughout Screening for DsbB Inhibitors

**Figure 6:**
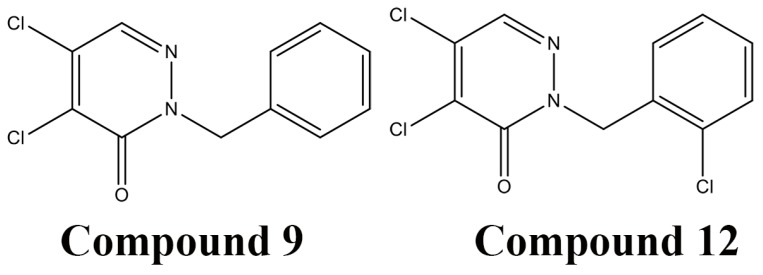
Structures of the most potent DsbB inhibitors identified by a high throughput screening method, compounds **9** (2-benzyl-4,5-dicholorpyridazin-3(2*H*)-one) and compound **12** (4,5-dichloro-2-(2-chlorobenzyl)pyridazin-3(2*H*)-one).

A cell-based high-throughput approach was developed to identify inhibitors of DsbB [80]. This method involved using *E. coli* cells expressing β-galactosidase in the periplasm (β-Gal^dbs^), which is inactivated upon acquisition of a disulfide bond catalysed by the DsbA/DsbB redox pathway. This screening procedure allowed a rapid identification and ranking of DsbB inhibitors based upon the blue color resulting from X-Gal hydrolysis by β-Gal^dbs^. The authors screened 51,487 compounds using *E. coli* cells expressing β-Gal^dbs^ which yielded 6 *Ec*DsbB inhibitors. These compounds were unable to inhibit a functionally related protein VKOR from *Mycobacterium tuberculosis*, when tested on an *E. coli Mtb*VKOR dependent β-Gal^dbs^ strain, indicating some specificity for EcDsbB. Each of the inhibitors shared a pyridazinone ring structure and initial SAR studies using commercial analogues led to more effective DsbB inhibitors (Figure 6), which also inhibited purified EcDsbB in a EcDsbB-mediated ubiquinone reduction assay. Notably, the identified inhibitors also displayed different levels of inhibition for DsbB enzymes from other important Gram-negative pathogens such as *Acinetobacter baumanni*, *Klebsiella pneumoniae, Vibrio cholera, Hemophilus influenza, Francisella tularensis, Pseudomonas aeruginosa* and *Salmonella* Typhimurium [80]. 

## 6. Conclusions 

Bacterial infections are one of the greatest health threats of the 21st century. With the increase of bacterial resistance and a lack of new drugs coming into the market, there is an urgent need for a coordinated global effort to stimulate the development of novel therapeutics to replace the current failing antibiotics. Interfering with bacterial virulence rather than survival provides a potentially useful but mostly unexplored new antibacterial strategy. This approach offers a wealth of theoretically druggable new targets and could yield antimicrobial agents that impose less selective pressure on bacteria, which is likely to reduce or delay the development of resistance. However, the development of any anti-virulence therapy requires target-specific *in vitro* and *in vivo* assays to screen for compounds that block the specific virulence phenotype. Furthermore, effective administration of anti-virulence therapies will necessitate developing real time diagnostics that identify the etiological agent.

Virulence pathways currently being targeted for the development of anti-virulence agents include the bacterial Dsb disulfide catalytic systems. These redox systems are widespread across bacteria where they mediate the correct folding of numerous virulence factors that are required at every stage of the infection process [14]. Specifically, the periplasmic disulfide catalyst DsbA and its cognate membrane protein DsbB have been associated with the production of a plethora of virulence factors in many bacterial pathogens and therefore represent potential virulence targets. Moreover, the structural conservation of DsbA proteins within different bacterial classes suggests that DsbA based anti-virulence agents could be effective against a wide range of pathogens. The suitability of this system as a therapeutic target is further substantiated by the low sequence identity of DsbAs to human oxidoreductase enzymes [81].

Targeting the bacterial DsbA/DsbB redox system is challenging, as there are difficulties associated with finding suitable high affinity binding locations alongside having to work with integral membrane proteins. Nevertheless, recent reports have described the use of different approaches, including fragment-based drug discovery, high-throughput screening and design of peptide and peptidomimetics, to develop inhibitors of the DsbA/DsbB oxidative system. FBDD approaches that combine state of the art technologies have yielded the first set of fragments that bind and inhibit the function of EcDsbB and EcDsbA in a dose-dependent manner, providing evidence that these important thiol-disulfide oxidoreductases are amenable to the development of antibacterial agents. Similarly, a cell-based high-throughput screening method yielded DsbB inhibitors that showed activity against DsbB homologues from different important human pathogens. Finally, peptides and peptidomimetics that inhibit DsbA proteins both in a reversible and irreversible manner have also been reported. To our knowledge there are no reported inhibitor development campaigns focused on the Dsb isomerase pathway. This pathway has received less attention, as it appears to have an overall lesser role in bacterial infection. An exception to this may be pathogenic strains of *Neisseria*, where it has been reported that DsbD is essential for viability, which offers the potential for the development of *Neisseria*-specific antibiotics [82].

Looking at the progress made with Dsb targeted therapies, we can say that the development of Dsb inhibitors is still in its infancy. To date all reported compounds exhibit weak potencies (high-micromolar to low millimolar), but encouragingly, they elicit phenotypes in bacterial cultures that are consistent with inhibiting the DsbA/DsbB redox system. These avenues to inhibit the Dsb mediated oxidative folding show promise for the development of anti-virulence agents and further investigation is required to discover their full potential. More potent compounds will reveal how Dsb inhibitors perform as anti-virulence drugs, whether they will be pathogen-specific molecules or show a broader spectrum of activity, and the rate and frequency at which resistance arises, which will impact whether they will be useful as monotherapy or in combination with standard antibiotics.

## Abbreviations

The following abbreviations are used in this manuscript:

CSP	Chemical Shift Perturbations
Dsb	Disulfide Bond
ESI-MS	Electrospray Ionization Mass Spectrometry
EPEC	Enteropathogenic *E. coli*
FBDD	Fragment-Based Drug Discovery
ITC	Isothermal Titration Calorimetry
LC-MS/MS	Liquid Chromatography-Mass Spectrometry
HSQC	Heteronuclear Single Quantum Coherence
MDR	Multi Drug Resistant
NMR	Nuclear Magnetic Resonance
Q	Quinones
SAR	Structure-Activity Relationship
TINS	Target Immobilized NMR Screening
TO	Terminal Oxidases
TR	Thioredoxin Reductase
UPEC	Uropathogenic *E. coli*
